# Usefulness of the Veterans Health Administration COVID-19 (VACO) Index for Predicting Short-Term Mortality among Patients of the COLOS Study

**DOI:** 10.3390/jcm12196262

**Published:** 2023-09-28

**Authors:** Agnieszka Matera-Witkiewicz, Magdalena Krupińska, Adrian Doroszko, Małgorzata Trocha, Katarzyna Giniewicz, Krzysztof Kujawa, Maciej Rabczyński, Marta Obremska, Edwin Kuznik, Pawel Lubieniecki, Barbara Adamik, Krzysztof Kaliszewski, Katarzyna Kiliś-Pstrusińska, Michał Pomorski, Marcin Protasiewicz, Marcin Madziarski, Janusz Sokołowski, Ewa A. Jankowska, Katarzyna Madziarska

**Affiliations:** 1 Screening of Biological Activity Assays and Collection of Biological Material Laboratory, Wroclaw Medical University Biobank, Faculty of Pharmacy, Wroclaw Medical University, Borowska Street 221A, 50-556 Wroclaw, Poland; 2 Clinical Department of Internal and Occupational Diseases, Hypertension and Clinical Oncology, Wroclaw Medical University, Borowska Street 213, 50-556 Wroclaw, Poland; adrian.doroszko@umw.edu.pl; 3 Clinical Department of Diabetology and Internal Disease, Wroclaw Medical University, Borowska Street 213, 50-556 Wroclaw, Polandmaciej.rabczynski@umw.edu.pl (M.R.); edwin.kuznik@usk.wroc.pl (E.K.); pawel.lubieniecki@usk.wroc.pl (P.L.); katarzyna.madziarska@umw.edu.pl (K.M.); 4 Statistical Analysis Centre, Wroclaw Medical University, K. Marcinkowski Street 2-6, 50-368 Wroclaw, Poland; katarzyna.giniewicz@umw.edu.pl (K.G.); krzysztof.kujawa@umw.edu.pl (K.K.); 5 Institute of Heart Diseases, Wroclaw Medical University, Borowska Street 213, 50-556 Wroclaw, Poland; marta.obremska@umw.edu.pl (M.O.); marcin.protasiewicz@umw.edu.pl (M.P.); ewa.jankowska@umw.edu.pl (E.A.J.); 6 Clinical Department of Anaesthesiology and Intensive Therapy, Wroclaw Medical University, Borowska Street 213, 50-556 Wroclaw, Poland; barbara.adamik@umw.edu.pl; 7 Clinical Department of General, Minimally Invasive and Endocrine Surgery, Wroclaw Medical University, Borowska Street 213, 50-556 Wroclaw, Poland; krzysztof.kaliszewski@umw.edu.pl; 8 Clinical Department of Paediatric Nephrology, Wroclaw Medical University, Borowska Street 213, 50-556 Wroclaw, Poland; katarzyna.kilis-pstrusinska@umw.edu.pl; 9 Clinical Department of Gynecology and Obstetrics, Wroclaw Medical University, Borowska Street 213, 50-556 Wroclaw, Poland; michal.pomorski@umw.edu.pl; 10 Clinical Department of Rheumatology and Internal Medicine, University Hospital, Borowska Street 213, 50-556 Wroclaw, Poland; marcin.madziarski@usk.wroc.pl; 11 Department of Emergency Medicine, Wroclaw Medical University, Borowska Street 213, 50-556 Wroclaw, Poland; janusz.sokolowski@umw.edu.pl

**Keywords:** COVID-19, VACO-index, SARS-CoV2, mortality

## Abstract

Advanced age is known to be a predictor with COVID-19 severity. Understanding of other disease progression factors may shorten the time from patient admission to applied treatment. The Veterans Health Administration COVID-19 (VACO index) was assumed to additionally anticipate clinical results of patients hospitalized with a proven infection caused by the SARS-CoV-2 virus. Methods: The medical records of 2183 hospitalized patients were retrospectively analyzed. Patients were divided into four risk-of-death categories: low risk, medium risk, high-risk, and extreme risk depending on their VACO index calculation. Results: Significant differences in the mortality at the hospital after three months of discharge and six months after discharge were noticed. For the patients in the extreme-risk group, mortality reached 37.42%, 62.81%, and 78.44% for in-hospital, three months of discharge, and six months of discharge, respectively. The mortality marked as high risk reached 20.38%, 37.19%, and 58.77%. Moreover, the secondary outcomes analysis acknowledged that patients classified as extreme risk were more likely to suffer from cardiogenic shock, myocardial infarction, myocardial injury, stroke, pneumonia, acute kidney injury, and acute liver dysfunction. Patients at moderate risk were more often admitted to ICU when compared to other patients. Conclusions: The usage of the VACO index, combined with an appropriate well-defined medical interview and past medical history, tends to be a helpful instrument in order to predict short-term mortality and disease progression based on previous medical records.

## 1. Introduction

The speed of the COVID-19 pandemic spread has determined a need to increase the efficiency of patient triage. More than 600 million cases have been noted with 6.7 million of deaths so far [1]. Some mathematical models related to the severity of COVID-19 were proposed [2]. The determination of accurate estimates is complex due to the lack of accurate measurements from the beginning of the infection. In most of the cases, patients admitted to the hospital were in advanced disease progression. Increasing the understanding of the factors influencing the risk of COVID-19 may prioritize treatment and prevent disease advancement [3]. The SARS-CoV-2 infection does not just determine the acute respiratory distress syndrome (ARDS), which begins a few days after the onset of disease symptoms [4]; this crucial fact was established by careful monitoring of the patient respiratory status. Research on SARS-CoV-2 infection was also focused on the involvement of the virus in cardiac failure. The outcomes from the ECHO-COVID study show the association between echocardiography phenotypes and in-hospital mortality [5]. Moreover, patients with proven left ventricular diastolic dysfunction (LVDD) were noticed to have poor outcomes when moved to the intensive care unit (ICU) as well as an increased death rate [6].

Patients who died due to the progression of COVID-19 disease had the presence of the intracellular virus in the lungs as well as disrupted cell membranes; moreover, the pulmonary vessels showed the widespread thrombosis and microangiopathy [7]. SARS-CoV-2 is also known to affect patients with acute kidney injury (AKI). The consequences of disease progression may arise and complicate up to a quarter of patients hospitalized with COVID-19 infection. The disease progression is being associated with an increased risk for both morbidity and death [8].

Although disease progression factors have been recognized, the clinical behavior of infection in individuals is nevertheless unclear.

VACO assesses pre-existing medical results from SARS-CoV-2 infection from the US Veterans Health Administration (VA) and was validated in two independent prospective samples giving the clinicians the possibility to correctly triage patients [9]. The index predicts 30-day all-cause mortality in patients with COVID-19. The regression model during the index development was based on parameters such as age, sex, race-specific clinical outcomes, and the Charlson comorbidity index (CCI) value. Validation of the index was based on comparison between the area under receiver operating characteristic curves (AUC) in validation cohorts and subgroups based on demographics and the geographic region of living. As well as previously being validated among US veterans, the VACO index was also assessed among Medicare inpatients and outpatients older that 65 years. The group consisted of 427,224 patients [10]. The VACO index was found to estimate the risk of short-term mortality of patients diagnosed to have COVID-19 using the medical data prior to or at the time of diagnosis [4].

The appropriate classifications of patients in the risk stratum may pick up the comprehension of the long term COVID-19 syndrome which is known to affect almost 45% of COVID-19 survivors [11]. The aim of this study is to propose a usage of the VACO index as a scale to potentially identify severe cases and propose effective treatment based on prior medical records in order to prevent the disease progression at its beginning.

## 2. Materials and Methods

### 2.1. Study Design and Population

In our study, the characteristics of 2183 patients with COVID-19 hospitalized at the University Hospital in Wroclaw between February 2020 and June 2021 were used. The study protocol was approved by the Bioethics Committee and Review Board at the Wroclaw Medical University, Wroclaw, Poland (No: KB-444/2021). All medical records were collected as a part of the Coronavirus in the Lower Silesia–COLOS study. The initial diagnosis of SARS-CoV-2 was confirmed by reverse transcription polymerase chain reaction (RT-PCR) for viral RNA of nasopharyngeal swab specimens. Demographic variables (age and sex) and medical conditions included individual components of the Charlson comorbidity index (CCI) as predictors for the VACO index were applied. After VACO index calculation, patients were divided to the separate groups in terms of their obtained result.

– Low risk (L) 0–0.292%;
– Medium risk (M) 0.293–8.784%;
– High risk (H) 8.785–20.174%;
– Extreme risk (E) > 20.174% [4].

### 2.2. Follow-Up and Outcomes

All the study participants had the clinical assessment during the hospital admission. The clinical assessment of patients involved evaluating their symptoms and physical findings. The vital signs were measured, including temperature, heart rate, respiratory rate, blood pressure, and oxygen saturation. Blood samples were collected from patients in dedicated time hospitalization. Blood samples were also collected for further scientific purposes according to the Bioethics Committee Positive Opinion based on broad consent (No. KB—541/2018, KB—794/2018, KB—666/2019) in BD Vacutainer SSTII Advance (for biobanking of serum aliquots 4 × 0.5 mL in −65 °C/−86 °C range storage conditions) and BD Vacutainer K2E EDTA (for biobanking of whole blood with DL buffer 2 × 0.4 mL KP, 2 × 0.4 mL DL; plasma 2 × 0.8 mL and precipitate 2 × 0.7 mL; all in −65 °C/−86 °C range storage conditions). The samples are stored in Wroclaw Medical University Biobank ISO 20387:2021-01 accreditation (BB 001 Polish Centre for Accreditation) with ISO 9001 certification (FS708094) related to IAF Sector 38. We collected clinical data during the hospitalization period and afterward, data regarding death were fulfilled up to 6 months. In the post-discharge period, data regarding death were collected up to 6 months. A report on deaths after discharge from hospitals was provided by the National Health Fund upon request.

### 2.3. Statistical Analysis

Record numbers and percentages representing the descriptive data for categorical and numerical variables are presented as a mean with a standard deviation range (minimum–maximum). A significance value of 0.05 was selected and used during the statistical analyses. A Chi-square test was used as an omnibus test for categorical variables where more than 5 cases were expected. On the other hand, a Fisher exact test was used for cases with a case number of less than 5. The Welch’s ANOVA was used for continuous variables in order to look for unequal variances between the studied groups. The Games-Howell test with Tukey correction was used as post hoc analysis for continuous variables. For categorical variables, a post hoc test and the omnibus test were the same, although performed in subgroups with Bonferroni correction.

Time-dependent ROC analysis with an inverse probability of censoring weighting (IPCW) estimation was performed for in-hospital and all-cause mortality analyses. The VACO index score was assessed as a time-dependent area under the curve (AUC). A log-rank test confirmed the differences in survival curves between the low-risk, medium-risk, high-risk, and extreme-risk groups. The Grambsch–Therneau test was used to verify a proportional hazard assumption. The hazard ratio (HR) for the VACO score, its components, and risk strata were analyzed using the Cox proportional hazard model. Due to the dichotomic nature of secondary outcomes, a logistic regression model was fitted. Predictive capabilities were assessed with the use of classical ROC analysis and an AUC measurement. All statistical analyses were performed with R version 4.0.4 using the packages time-ROC, pROC [12], survival [13], and Coin [14]. Table abbreviations: OMNIBUS—analysis of variance, N—valid measurements, n—number of patients with a parameter above the cut-off point, SD—standard deviation, BMI—body mass index, DM—diabetes mellitus, TIA—transient ischemic attack, COPD—chronic obstructive pulmonary disease, and N/A—non-applicable. Bold text refers to statistically significant values.

## 3. Results

### 3.1. Demographic Information of the COLOS Population

The baseline is presented in Table 1. The COLOS population is based on 2183 patients hospitalized at the University Hospital in Wroclaw. Patients were divided into one of four groups taking into account the VACO index calculated at the admission. The largest numerical force present is the moderate-risk (M) group (710 cases) with 27–32% of over 65 years old representatives. Moreover, these patients also had an elevated level of BMI > 30. Overall, 83–92% of elderly patients (over 65 years) were categorized as high-risk (H) patients (628 cases). Patients at extreme risk (326 cases) consisted only within the age group of more than 65. The highest prevalence of comorbidities such as hypertension, dyslipidemia, atrial fibrillation/flutter, heart failure, and previous coronary revascularization was observed. Due to concomitant diseases, patients from the extreme-risk group were more often given under the treatment before hospitalization. Furthermore, this group of patients was more often given angiotensin-converting-enzyme inhibitors (ACE), mineralocorticoid receptor antagonists (MRA), b-blockers, diuretics, statins, and vitamin K antagonists (VKA) due to previous stroke history and higher prevalences of cardiovascular diseases. On the other hand, patients in the high-risk stratum more often received new oral anticoagulants (NOAC), such as metformin. Patients at moderate risk were more often given oral corticosteroids and immunosuppression other than oral corticosteroids. All the data regarding treatment applied before hospitalization are shown in Table 2.

Patients in the low-risk group had a significantly higher prevalence of a cough, smell dysfunction, chest pain, and abdominal pain on admission. On the other hand, patients in the extreme-risk stratum had the highest prevalence of dyspnea, crackles, wheezing, pulmonary congestion, and peripheral edema detected during physical examination. All patient-reported symptoms, vital signs, and abnormalities measured during a physical examination at hospital admission are summarized in Table 3.

### 3.2. Laboratory Assays

All laboratory parameters are presented in Table 4. The extreme-risk group was characterized by the lowest level of hemoglobin and blood platelet count; on the other hand, these patients had higher potassium ion concentrations with coexisting elevated INR as well as higher serum levels of urea and creatinine coexisting with lower eGFR and albumin values. Also, significantly higher cardiac biomarkers (BNP, NT-proBNP, and troponin) have been recognized. Patients from the extreme-risk group also had the highest level of inflammatory biomarkers (CRP, D-dimer, and IL-6) compared to patients from other groups. Moderate-risk stratum patients had the highest platelet count as well as the lowest IL-6 level when compared to patients from other groups. Also, the highest LDH and HDL-cholesterol level were determined.

**Table 1:** COLOS cohort description after VACO risk stratification.

Variables, Units (N)	Lower Risk (L) [0–0.292%]	Moderate Risk (M) [0.293–8.784%]	High Risk (H) [8.785–20.174%]	Extreme Risk (E) [>20.174%]	OMNIBUS *p-*Value		
	Mean ± SD Min–Max (N) or n/N (% of Risk Category)	Mean ± SD Min–Max (N) or n/N (% of Risk Category)	Mean ± SD Min–Max (N) or n/N (% of Risk Category)	Mean ± SD Min–Max (N) or n/N (% of Risk Category)			
						a—L vs. M b—L vs. H c—L vs. E	d—M vs. H e—M vs. E f—H vs. E
	Demographics						
**Age, years** (2183)	34.36 ± 8.22 17–49 (519)	58.08 ± 11.1 17–79 (710)	70.94 ± 6.45 57–84 (628)	84.37 ± 6.64 69–100 (326)	**<0.0001**	**<0.0001 a,b,c**	**<0.0001 d,e,f**
**Age ≥ 65 years** (2183)	0/519 (0.0%)	194/710 (27.32%)	527/628 (83.92%)	326/326 (100%)	**<0.0001**	**<0.0001 a,b,c**	**<0.0001 d,e,f**
**Male gender** (2183)	201/519 (38.73%)	352/710 (49.58%)	348/628 (55.41%)	181/326 (55.52%)	**<0.0001**	**0.0012 a** **<0.0001 b,c**	0.2261 d 0.5228 e 1.0 f
**BMI kg/m^2^** (554)	27.93 ± 5.54 15.36–49.38 (170)	29.22 ± 5.34 17.07–48.21 (187)	28.25 ± 5.0 17.11–45.82 (137)	27.36 ± 4.62 16.41–37.04 (60)	**0.0357**	0.117 a 0.953 b 0.86 c	0.336 d **0.049** e 0.619 f
**BMI kg/m^2^ > 30** (554)	49/170 (28.82%)	73/187 (39.04%)	50/137 (36.5%)	19/60 (31.67%)	**0.0195**	**0.0229 a** 1.0 b,c	0.3948 d 0.2208 e 1.0 f
**Cigarette smoking:** **Never;** **Previous;** **Current** (2183)	504/519 (97.11%) 6/519 (1.16%) 9/519 (1.73%)	660/710 (93.22%) 28/710 (3.95%) 20/710 (2.82%)	533/628 (85.14%) 57/628 (9.11%) 36/628 (5.75%)	289/326 (88.65%) 26/326 (7.98%) 11/326 (3.37%)	**<0.0001**	**0.0323 a** **<0.0001 b,c**	**<0.0001 d** 0.1314 e 1.0 f
**Co-morbidities**							
**Hypertension** (2183)	38/519 (7.32%)	289/710 (40.7%)	445/628 (70.86%)	249/326 (76.38%)	**<0.0001**	**<0.0001 a,b,c**	**<0.0001 d,e** 0.4916 f
**DM** (2183)	0/519 (0.0%)	90/710 (12.68%)	254/628 (40.51%)	128/326 (39.38%)	**<0.0001**	**<0.0001 a,b,c**	**<0.0001** d,e 1.0 f
**Dyslipidemia** (825)	43/65 (66.15%)	172/251 (68.53%)	250/334 (77.25%)	138/175 (78.86%)	**0.0188**	1.0 a 0.4877 b 0.3753 c	0.139 d 0.1482 e 1.0 f
**Atrial fibrillation/flutter** (2183)	1/519 (0.19%)	32/710 (4.51%)	127/628 (20.22%)	130/326 (39.88%)	**<0.0001**	**<0.0001 a,b,c**	**<0.0001 d,e,f**
**Previous coronary revascularisation** (2183)	0/519 (0.0%)	7/710 (0.99%)	66/628 (10.51%)	81/326 (24.85%)	**<0.0001**	0.3567 a **<0.0001 b,c**	**<0.0001 d,e,f**
**Previous myocardial infarction** (2183)	0/519 (0.0%)	16/710 (2.25%)	90/628 (14.33%)	85/326 (26.07%)	**<0.0001**	**0.0086 a** **<0.0001 b,c**	**<0.0001 d,e,f**
**Heart failure** (2183)	0/519 (0.0%)	20/710 (2.82%)	106/628 (16.88%)	129/326 (39.57%)	**<0.0001**	**0.0017 a** **<0.0001 b,c**	**<0.0001 d,e,f**
**Moderate/severe valvular heart disease or previous valve heart surgery** (2183)	3/519 (0.58%)	12/710 (1.69%)	35/628 (5.57%)	46/326 (14.11%)	**<0.0001**	0.8161 a **<0.0001 b,c**	**0.0013 d** **<0.0001 e,f**
**Peripheral artery disease** (2183)	0/519 (0.0%)	11/710 (1.55%)	35/628 (5.57%)	54/326 (16.56%)	**<0.0001**	0.0662 a **<0.0001 b,c**	**0.0006 d** **<0.0001 e,f**
**Previous stroke/TIA** (2183)	0/519 (0.0%)	21/710 (2.96%)	73/628 (11.62%)	70/326 (21.47%)	**<0.0001**	**0.0012 a** **<0.0001 b, c**	**<0.0001 d,e** **0.0005 f**
**Chronic kidney disease** (2183)	0/519 (0.0%)	46/710 (6.48%)	90/628 (14.33%)	95/326 (29.14%)	**<0.0001**	**<0.0001 a,b,c**	**<0.0001 d,e,f**
**Hemodialysis** (2183)	0/519 (0.0%)	17/710 (2.39%)	27/628 (4.3%)	14/326 (4.29%)	**<0.0001**	**0.0057 a** **<0.0001 b,c**	0.4346 d 0.8483 e 1.0 f
**Asthma** (2183)	16/519 (3.08%)	39/710 (5.49%)	23/628 (3.66%)	7/326 (2.15%)	**0.0365**	0.3617 a 1.0 b 1.0 c	0.8668 d 0.1409 e 1.0 f
**COPD** (2183)	0/519 (0.0%)	4/710 (0.56%)	47/628 (7.48%)	24/326 (7.36%)	**<0.0001**	1.0 a **<0.0001 b,c**	**<0.0001 d,e** 1.0 f
**Thyroid disease** (2183)	51/519 (9.83%)	64/710 (9.01%)	84/628 (13.38%)	30/326 (9.2%)	**0.0453**	1.0 a 0.4658 b 1.0 c	0.0854 d 1.0 e 0.4509 f

Continuous variables are defined as mean ± SD, range (min–max), and non-missing number values. Categorized variables are shown as percentage numbers.

**Table 2:** Baseline characteristics of the study cohort; treatment applied before hospitalization.

Variables, Units (N)	Lower Risk (L) [0–0.292%]	Moderate Risk (M) [0.293–8.784%]	High Risk (H) [8.785–20.174%]	Extreme Risk (E) [>20.174%]	OMNIBUS *p-*Value	*p-*Value (for Post-Hoc Analysis)	
	n/N (% of Risk Category)	n/N (% of Risk Category)	n/N (% of Risk Category)	n/N (% of Risk Category)		a—L vs. M b—L vs. H c—L vs. E	d—M vs. H e—M vs. E f—H vs. E
**Treatment Applied Before Hospitalization**							
**ACEI angiotensin-converting-enzyme inhibitors** (2183)	3/519 (0.58%)	83/710 (11.69%)	165/628 (26.27%)	101/326 (30.98%)	**<0.0001**	**<0.0001 a,b,c**	**<0.0001 d,e** 0.8627 f
**ARB angiotensin receptor blockers** (2183)	8/519 (1.54%)	56/710 (7.89%)	51/628 (8.12%)	29/326 (8.9%)	**<0.0001**	**<0.0001 a,b,c**	1.0 d,e,f
**MRA mineralocorticoid receptor antagonists** (2183)	1/519 (0.0%)	17/710 (2.39%)	51/628 (8.12%)	32/326 (9.82%)	**<0.0001**	**0.0057 a** **<0.0001 b,c**	**<0.0001** e,f 1.0 f
**β-blocker** (2183)	15/519 (2.89%)	129/710 (18.17%)	250/628 (39.81%)	139/326 (42.64%)	**<0.0001**	**<0.0001 a,b,c**	**<0.0001 d,e** 1.0 f
**Digitalis glycoside** (2183)	0/519 (0.0%)	1/710 (0.14%)	11/628 (1.75%)	7/326 (2.14%)	**<0.0001**	1.0 a **0.0086 b** **0.0073 c**	**0.0125 d** **0.0102 e** 1.0 f
**Calcium channel blocker** **(non-dihydropiridines)** (2183)	2/519 (0.39%)	7/710 (0.99%)	19/628 (3.03%)	10/326 (3.07%)	0.0006	1.0 a **0.0117 b** **0.0218 c**	0.0748 d 0.173 e 1.0 f
**Calcium channel blocker** **(dihydropiridines)** (2183)	8/519 (1.54%)	71/710 (10.0%)	111/628 (17.68%)	71/326 (21.78%)	**<0.0001**	**<0.0001 a,b,c**	**<0.0001 d,e** 0.8937 f
**α-adrenergic blocker** (2183)	3/519 (0.58%)	19/710 (2.68%)	57/628 (9.08%)	39/326 (11.96%)	**<0.0001**	0.07 a **<0.0001 b,c**	**<0.0001 d,e** 1.0 f
**Thiazide or thiazide-like diuretic** (2183)	2/519 (0.39%)	50/710 (7.04%)	62/628 (9.87%)	36/326 (11.04%)	**<0.0001**	**<0.0001 a,b,c**	0.4636 d 0.2444 e 1.0 f
**Loop diuretic** (2183)	0/519 (0.0%)	26/710 (3.66%)	89/628 (14.17%)	70/326 (21.47%)	**<0.0001**	**0.0002 a** **<0.0001 b,c**	**<0.0001 d,e** **0.0328 f**
**Statin** (2183)	1/519 (0.19%)	62/710 (8.73%)	176/628 (28.03%)	111/326 (34.05%)	**<0.0001**	**<0.0001 a,b,c**	**<0.0001 d,e** 0.3862 f
**Acetylsalicylic acid** (2183)	9/519 (1.73%)	39/710 (5.49%)	136/628 (21.66%)	74/326 (22.7%)	**<0.0001**	**0.0079 a** **<0.0001 b,c**	**<0.0001 d,e** 1.0 f
**LMWH low molecular weight heparin** (2183)	16/519 (3.08%)	35/710 (4.93%)	68/628 (10.83%)	22/326 (6.75%)	**<0.0001**	0.8681 a **<0.0001** b 0.1181 c	**0.0005 d** 1.0 e 0.3233 f
**VKA vitamin K antagonists** (2183)	1/519 (0.19%)	9/710 (1.27%)	23/628 (3.66%)	53/326 (16.26%)	**<0.0001**	0.4802 a **0.0006 b** **0.0002 c**	**0.0439 d** **0. 0268 e** 1.0 f
**NOAC novel oral anticoagulants** (2183)	0/519 (0.0%)	11/710 (1.55%)	43/628 (6.85%)	14/326 (4.29%)	**<0.0001**	0.0662 a **<0.0001 b,c**	**<0.0001 d,e,f**
**Insulin** (2183)	3/519 (0.58%)	28/710 (3.94%)	62/628 (9.87%)	38/326 (11.66%)	**<0.0001**	**0.0025 a** **<0.0001 b,c**	**0.0002 d** **<0.0001 e** 1.0 f
**Metformin** (2183)	1/519 (0.19%)	42/710 (5.92%)	129/628 (20.54%)	50/326 (15.34%)	**<0.0001**	**<0.0001 a,b,c**	**<0.0001 d,e** 0.3729 f
**SGLT2 inhibitor inhibitors—sodium glucose co-transporter-2 inhibitors** (2183)	0/519 (0.0%)	7/710 (0.99%)	15/628 (2.39%)	5/326 (1.53%)	**0.0001**	0.1413 a **0.0007 b** 0.0503 c	0.3156 d **1.0** e,f
**Oral antidiabetics other than SGLT2 inhibitor and metformin** (2183)	0/519 (0.0%)	4/710 (0.56%)	52/628 (8.28%)	33/326 (10.12%)	**0.0001**	1.0 a 0.0001 b, c	0.0001 d,e 1.0 f
**Proton pump inhibitor** (2183)	6/519 (1.16%)	58/710 (8.17%)	111/628 (17.68%)	75/326 (23.01%)	**0.0001**	**0.0001 a,b,c**	**0.0001 d,e** 0.3565 f
**Oral corticosteroid** (2183)	4/519 (0.77%)	46/710 (6.48%)	34/628 (5.41%)	8/326 (2.45%)	**0.0001**	**0.0001 a** **0.0002 b** 0.5187 c	1.0 d **0.0635** e 0.309 f
**Immunosuppression other than oral corticosteroid** (2183)	2/519 (0.39%)	37/710 (5.21%)	30/628 (4.78%)	4/326 (1.23%)	**0.0001**	**0.0001** a,b 1.0 c	1.0 d **0.0236 e** 0.0526 f

Categorized variables are shown as a number with a percentage number. Information about the numbers with valid values is provided in the left column.

**Table 3:** Patient-reported symptoms, vital signs, and abnormalities measured during physical examination at hospital admission in the study cohort after VACO risk stratification.

Variables, Units (N)	Lower Risk (L) [0–0.292%]	Moderate Risk (M) [0.293–8.784%]	High Risk (H) [8.785–20.174%]	Extreme risk (E) [>20.174%]	OMNIBUS *p-*Value	*p-*Value (for Post-Hoc Analysis)	
	Min–Max (N) or n/N (% of Risk Category)	Min–Max (N) or n/N (% of Risk Category)	Min–Max (N) or n/N (% of Risk Category)	Min–Max (N) or n/N (% of Risk Category)		a—L vs. M b—L vs. H c—L vs. E	d—M vs. H e—M vs. E f—H vs. E
**Patient-Reported Symptoms**							
**Cough** (2183)	193/519 (37.19%)	236/710 (33.24%)	148/628 (23.57%)	71/326 (21.78%)	**<0.0001**	1.0 a **<0.0001 b,c**	**0.0007 d** **0.0014** e 1.0 f
**Dyspnea** **(2183)**	181/519 (34.87%)	342/710 (48.17%)	245/628 (39.01%)	153/326 (46.93%)	**<0.0001**	**<0.0001** a 1.0 b **0.0038 c**	**0.0055 d** 1.0 e 0.1344 f
**Chest pain** **(2183)**	49/519 (9.44%)	51/710 (7.18%)	35/628 (5.57%)	28/326 (8.59%)	0.0764	N/A	N/A
**Hemoptysis** (2183)	2/519 (0.39%)	7/710 (0.99%)	4/628 (0.64%)	2/326 (0.61%)	0.6784	N/A	N/A
**Smell dysfunction** **(2183)**	30/519 (5.78%)	29/710 (4.08%)	10/628 (1.59%)	7/326 (2.15%)	**0.0006**	1.0 a **0.0014 b** 0.1158 c	0.0662 d 0.9719 e 1.0 f
**Taste dysfunction** **(2183)**	19/519 (3.66%)	30/710 (4.23%)	11/628 (1.75%)	6/326 (1.84%)	**0.0263**	1.0 a 0.4028 b 1.0 c	0.0831 d 0.4667 e 1.0 f
**Abdominal pain** (2183)	41/519 (7.9%)	49/710 (6.9%)	40/628 (6.4%)	19/326 (4.91%)	0.387	N/A	N/A
**Diarrhea** **(2183)**	20/519 (3.85%)	41/710 (5.77%)	48/628 (7.64%)	18/326 (5.52%)	0.0568	N/A	N/A
**Nausea/Vomiting** **(2183)**	17/519 (3.28%)	29/710 (4.08%)	36/628 (5.73%)	16/326 (4.91%)	0.2167	N/A	N/A
**Measured vital signs**							
**Body temperature °C** (1185)	37.13 ± 0.88 35.4–40.0 (337)	37.06 ± 0.9 34.4–40.5 (389)	36.91 ± 0.87 35.2–40.0 (309)	36.92 ± 0.88 35.0–40.0 (150)	**0.0055**	0.774 a **0.009 b** 0.077 c	0.104 d 0.324 e 1.0 f
**Heart rate** **beats/minute** (1672)	87.84 ± 14.96 48–160 (373)	85.38 ± 15.22 48–150 (538)	84.41 ± 17.46 50–170 (492)	85.36 ± 17.93 36–150 (269)	**0.0144**	0.073 a **0.011 b** 0.25 c	0.782 d 1.0 e 0.9 f
**Respiratory rate breaths/minute** (318)	17.65 ± 4.64 12–40 (77)	18.88 ± 6.01 12–50 (97)	18.71 ± 6.62 12–50 (95)	19.06 ± 4.92 12–31 (49)	0.3055	N/A	N/A
**Systolic blood pressure** (1669)	125.17 ± 16.77 74–200 (364)	134.78 ± 22.08 60–240 (534)	133.4 ± 23.75 50–237 (500)	133.64 ± 27.53 50–270 (271)	**<0.0001**	**<0.0001 a,b,c**	0.77 d 0.935 e 0.999 f
**Diastolic blood pressure** **(1661)**	77.86 ± 11.33 48–120 (364)	79.37 ± 12.52 40–120 (533)	77.62 ± 14.29 40–150 (496)	76.33 ± 15.35 40–157 (268)	**0.0213**	0.235 a 0.993 b 0.516 c	0.159 d **0.027** e 0.667 f
**SpO_2_ on room air, % (FiO_2_ = 21%)** (1262)	95.22 ± 5.51 50–100 (344)	90.84 ± 8.01 48–100 (389)	90.68 ± 8.48 50–99 (344)	89.44 ± 9.39 50–100 (185)	**<0.0001**	**<0.0001 a,b,c**	0.993 d 0.303 e 0.445 f
**Abnormalities detected during physical examination**							
**Crackles** **(2183)**	30/519 (5.78%)	101/710 (14.23%)	119/628 (18.95%)	69/326 (21.17%)	**<0.0001**	**<0.0001 a,b,c**	0.1457 d **0.0403** e 1.0 f
**Wheezing** **(2183)**	18/519 (3.47%)	52/710 (7.32%)	90/628 (14.33%)	59/326 (18.1%)	**<0.0001**	**0.0351 a** **<0.0001 b,c** 0.86 c	**0.0003 d** **<0.0001** e 0.9231 f
**Pulmonary congestion** **(2183)**	34/519 (6.55%)	120/710 (16.9%)	126/628 (20.06%)	87/326 (26.69%)	**<0.0001**	**<0.0001 a,b,c**	0.9344 d **0.0021** e 0.1475 f
**Peripheral edema** (2183)	6/519 (1.16%)	58/710 (8.17%)	80/628 (12.74%)	45/326 (13.8%)	**<0.0001**	**<0.0001 a,b,c**	**0.0479 d** **0.0412** e 1.0 f

Continuous variables are shown as mean ± SD, range (min–maxi), and number of non-missing values. Categorized variables are presented as percentages. Information about the numbers with valid values is provided in the left column. N/A: Not applicable.

**Table 4:** Laboratory parameters of the COLOS cohort.

Parameter (N)	Time of Assessment	Units	Lower Risk (L) [0–0.292%]	Moderate Risk (M) [0.293–8.784%]	High Risk (H) [8.785–20.174%]	Extreme Risk (E) [>20.174%]	OMNIBUS *p*-Value	*p*-Value (for Post-Hoc Analysis)	
			Mean ± SD Min–Max (N) or n/N (% of Risk Category)	Mean ± SD Min–Max (N) or n/N (% of Risk Category)	Mean ± SD Min–Max (N) or n/N (% of Risk Category)	Mean ± SD Min–Max (N) or n/N (% of Risk Category)		a—L vs. M b—L vs. H c—L vs. E	d—M vs. H e—M vs. E f—H vs. E
**Complete Blood Count (CBC)**									
**Leucocytes** (2048)	**On admission**	×10^3^/µL	8.32 ± 8.58 1.76–163.61 (443)	8.7 ± 12.56 0.67–304.02 (675)	9.43 ± 10.98 0.51–188.7 (615)	10.46 ± 14.31 0.51–215.97 (315)	0.0644	N/A	N/A
**Hemoglobin** (2048)	**On admission**	g/dL	13.44 ± 1.94 4.8–18.7 (443)	13.31 ± 2.11 4.3–17.9 (675)	12.59 ± 2.42 3.9–20.3 (615)	12.11 ± 2.46 4.5–18.9 (315)	**<0.0001**	0.706 **a** **<0.0001 b,c**	**<0.0001 d,e** **0.024 f**
**Platelets** (2048)	**On admission**	×10^3^/µL	221.19 ± 86.73 23.0–811.0 (443)	240.62 ± 114.64 4.0–1356.0 (675)	238.46 ± 116.79 0.0–879.0 (615)	215.86 ± 99.89 3.0–667.0 (315)	**0.0002**	**0.007 a** **0.03 b** 0.871 c	0.987 **d** **0.003 e** **0.012 f**
**Acid–base balance in arterial blood gas**									
**PH** **(276)**	**On admission**		7.4 ± 0.09 7.16–7.5 (14)	7.43 ± 0.07 7.19–7.57 (72)	7.43 ± 0.07 7.04–7.58 (104)	7.42 ± 0.09 7.09–7.54 (86)	0.5873	N/A	N/A
**PaO_2_** (276)	**On admission**	mmHg	72.34 ± 16.38 43.3–96.7 (14)	73.95 ± 31.64 12.8–100.0 (72)	71.71 ± 36.48 26.8–100.0 (104)	74.34 ± 42.7 23.7–100.0 (86)	0.9605	N/A	N/A
**PaCO_2_** **(276)**	**On admission**	mmHg	43.53 ± 14.07 25.8–80.9 (14)	37.58 ± 10.44 20.2–82.4 (72)	37.35 ± 10.04 20.09–88.4 (104)	37.38 ± 9.22 19.7–74.9 (86)	0.475	N/A	N/A
**HCO3 standard** **(272)**	**On admission**	mmol/L	25.27 ± 3.6 18.9–31.3 (14)	24.83 ± 3.15 12.5–31.7 (71)	24.51 ± 3.82 14.3–32.9 (103)	24.26 ± 5.14 12.1–39.5 (84)	0.7442	N/A	N/A
**BE** (108)	**On admission**	mmol/L	2.9 ± 0.99 2.2–3.6 (2)	0.7 ± 4.85 [-]15.7–7.9 (27)	0.79 ± 5.15 12.5–10.5 (44)	2.89 ± 5.04 [-]4.3–15.7 (35)	0.1668	N/A	N/A
**Lactates** (245)	**On admission**	mmol/L	2.25 ± 0.74 0.7–3.3 (13)	2.34 ± 1.12 0.6–5.9 (60)	2.29 ± 1.31 0.5–10.1 (95)	2.38 ± 1.97 0.5–12.8 (77)	0.9717	N/A	N/A
**Electrolytes and inflammatory and iron biomarkers**									
**Na** **(2030)**	**On admission**	mmol/L	138.46 ± 2.95 128.6–148.0 (439)	138.23 ± 4.6 109.0–158.0 (666)	137.35 ± 6.43 101.0–175.0 (610)	138.75 ± 7.25 105.0–174.0 (315)	**0.0014**	0.759 a **0.001 b** 0.910 c	**0.026 d** 0.646 e **0.02 f**
**K** **(2037)**	**On admission**	mmol/L	3.99 ± 0.44 2.7–5.4 (440)	4.05 ± 0.61 2.4 ± 6.57 (670)	4.18 ± 0.7 2.0–7.5 (613)	4.25 ± 0.81 2.4–8.7 (314)	**<0.0001**	0.247 a **<0.0001 b,c**	**0.002 d** **0.0009 e** 0.611 f
**CRP** **(2018)**	**On admission**	mg/L	49.79 ± 70.34 0.34–390.8 (425)	86.06 ± 84.21 0.13–531.58 (669)	82.77 ± 84.56 0.29–496.98 (610)	90.02 ± 94.63 0.4–538.55 (314)	**<0.0001**	**<0.0001 a,b,c**	0.898 d 0.922 e 0.664 f
	**On discharge**		26.96 ± 56.53 0.28–452.93 (425)	51.48 ± 80.03 0.13–494.73 (669)	70.77 ± 92.94 0.22–496.98 (610)	85.25 ± 95.26 0.4–538.55 (314)	**<0.0001**	**<0.0001 a,b,c**	**0.0005 d** **<0.0001 e** 0.123 f
**Procalcitonin** (1473)	**On admission**	ng/mL	0.21 ± 0.95 0.01–10.51 (283)	0.58 ± 2.57 0.01–44.39 (497)	1.32 ± 5.42 0.01–55.01 (454)	3.2 ± 15.58 0.01–196.04 (239)	**<0.0001**	**0.023 a** **0.0002 b** **0.018 c**	**0.04 d** 0.051 **e** 0.274 f
**IL-6** (701)	**On admission**	pg/mL	87.49 ± 756.98 2.0–9099.0 (144)	40.94 ± 84.32 2.0–1000.0 (279)	53.79 ± 96.09 2.0–1000.0 (205)	70.53 ± 140.37 2.0–1000.0 (82)	0.1682	N/A	N/A
**D-dimer** **(1578)**	**On admission**	µg/mL	2.13 ± 9.04 0.15–128.0 (323)	4.24 ± 13.27 0.21–123.93 (538)	5.85 ± 16.62 0.18–132.82 (485)	6.27 ± 15.82 0.2–128.0 (232)	**<0.0001**	**0.029 a** **0.0003 b** **0.002 c**	**<0.0001 d,e,f**
**INR** **(1923)**	**On admission**		1.05 ± 0.14 0.82–2.26 (416)	1.16 ± 0.34 0.83–7.14 (628)	1.33 ± 1.17 0.87–18.74 (583)	1.56 ± 1.92 0.89–21.1 (296)	**<0.0001**	**<0.0001 a,b,c**	**0.008 d** **0.003 e** 0.213 f
**Fibrinogen** **(419)**	**On admission**	g/dL	4.86 ± 1.52 0.64–9.04 (126)	4.8 ± 2.13 0.35–10.0 (127)	4.72 ± 1.78 0.37–9.2 (120)	4.95 ± 1.8 2.1–9.1 (46)	0.8748	N/A	N/A
**Biochemistry**									
**Glucose** (1758)	**On admission**	mg/dL	109.22 ± 26.04 50.0–247.0 (272)	133.82 ± 69.3 28.0–933.0 (624)	160.11 ± 103.85 35.0–1064.0 (568)	149.96 ± 90.74 47.0–1026.0 (294)	**<0.0001**	**<0.0001 a,b,c**	**<0.0001 d** **0.036 e** 0.45 f
**Glycated hemoglobin (HbA1 c)** (263)	**On admission**	%	5.743 ± 1.46 4.2–10.8 (16)	7.57 ± 2.25 4.8–14.9 (63)	7.81 ± 2.18 4.6–16.6 (116)	7.36 ± 1.99 5.1–14.9 (68)	**<0.0001**	**0.002 a** **0.0002 b** **0.004 c**	0.901 d 0.944 **e** 0.488 f
**Urea** **(1857)**	**On admission**	mg/dL	24.26 ± 12.02 5.0–81.0 (331)	44.35 ± 33.41 10.0–336.0 (634)	63.08 ± 48.33 8.0–353.0 (592)	82.46 ± 54.3 12.0–369.0 (300)	**<0.0001**	**<0.0001 a,b,c**	**<0.0001 d,e,f**
**Creatinine** (1961)	**On admission**	mg/dL	0.81 ± 0.22 0.26–2.23 (364)	1.2 ± 1.24 0.34–14.87 (669)	1.51 ± 1.49 0.35–14.77 (613)	1.76 ± 1.47 0.46–10.84 (315)	**<0.0001**	**<0.0001 a,b,c**	**0.0003 d** **<0.0001 e** 0.077 f
	**On discharge**		0.79 ± 0.2 0.26–1.67 (364)	1.15 ± 1.17 0.34–14.87 (669)	1.4 ± 1.35 0.36–14.82 (613)	1.67 ± 1.46 0.43–9.27 (315)	**<0.0001**	**<0.0001 a,b,c**	**0.003 d** **<0.0001 e** **0.026 f**
**eGFR** (1956)	**On admission**	mL/min/1.73 m^2^	105.21 ± 29.9 0.0–433.0 (361)	78.31 ± 30.46 3.0–250.0 (667)	65.48 ± 32.88 3.0–239.0 (613)	51.7 ± 26.62 5.0–139.0 (315)	**<0.0001**	**<0.0001 a,b,c**	**<0.0001 d,e,f**
	**On discharge**		107.07 ± 29.95 0.0–433.0 (361)	82.38 ± 32.5 4.0–239.0 (667)	70.88 ± 34.69 3.0–323.0 (613)	57.2 ± 30. 4.0–170.0 (315)	**<0.0001**	**<0.0001 a,b,c**	**<0.0001 d,e,f**
**Total protein** (606)	**On admission**	g/L	6.23 ± 0.72 4.2–8.0 (82)	6.0 ± 0.9 3.5–8.2 (189)	5.97 ± 0.86 3.8–9.5 (209)	5.74 ± 0.92 3.3–8.2 (126)	0.0005	0.121 a 0.051 **b** **0.0002 c**	0.99 d 0.068 **e** 0.104 f
**Albumin** (663)	**On admission**	g/L	3.35 ± 0.61 1.7–5.1 (90)	3.1 ± 0.6 1.5–4.5 (212)	3.12 ± 0.52 1.7–4.4 (236)	2.95 ± 0.66 0.7–4.9 (125)	**0.0001**	**0.006 a** **0.013 b** **<0.0001 c**	0.957 d 0.165 **e** 0.051 f
**AST** (1441)	**On admission**	IU/L	48.88 ± 47.24 9.0–347.0 (257)	62.58 ± 128.08 6.0–2405.0 (486)	59.63 ± 94.44 5.09–83.0 (466)	101.94 ± 435.2 8.0–4776.0 (232)	**0.022**	0.153 a 0.175 **b** 0.254 c	0.977 d 0.532 **e** 0.461 f
**ALT** **(1588)**	**On admission**	IU/L	46.11 ± 57.82 7.0–591.0 (285)	57.44 ± 99.06 5.0–1411.0 (536)	46.95 ± 76.64 4.0–854.0 (507)	63.47 ± 262.41 5.0–3700.0 (260)	0.1276	N/A	N/A
**Bilirubin** **(1407)**	**On admission**	mg/dL	0.66 ± 0.63 0.2–6.7 (232)	0.83 ± 1.48 0.1–19.1 (481)	0.91 ± 1.25 0.2–15.1 (461)	0.91 ± 0.89 0.1–9.2 (233)	**0.0002**	0.122 a **0.002 b,c**	0.788 d 0.793 **e** 1.0 f
**LDH** **(1231)**	**On admission**	U/L	378.2 ± 239.64 120.0–1720.0 (217)	441.04 ± 381.52 50.0–7100.0 (452)	419.81 ± 342.08 44.0–4107.0 (384)	436.29 ± 713.82 141.0–9505.0 (178)	0.0635	N/A	N/A
**Cardiac biomarkers**									
**BNP** (359)	**On admission**	pg/mL	39.06 ± 43.86 2.5–158.7 (18)	309.74 ± 785.23 1.7–4993.0 (94)	507.34 ± 1381.13 3.0–13,368.4 (150)	762.63 ± 1504.61 25.6–11,275.7 (97)	**<0.0001**	**0.007 a** **0.0003 b** **<0.0001 c**	0.486 d **0.047 e** 0.536 f
**NT-proBNP** (379)	**On admission**	ng/mL	349.31 ± 721.06 23.6–3577.0 (26)	3381.67 ± 11,223.05 12.0–70,000.0 (132)	7764.16 ± 14,150.85 29.7–70,000.0 (133)	13,015.59 ± 18,325.17 269.7–70,000.0 (88)	**<0.0001**	**0.014 a** **<0.0001 b,c**	**0.028 d** **0.0001 e** 0.108 f
**Troponin I** **(1174)**	**On admission**	pg/mL	29.42 ± 168.33 0.0–1789.6 (167)	86.76 ± 400.95 1.0–5120.4 (407)	867.12 ± 6995.2 0.2–125,592.6 (389)	1806.29 ± 10,669.28 2.3–109,359.5 (211)	**0.0008**	0.076 a 0.087 **b** 0.077 c	0.126 d 0.092 **e** 0.658 f
	>3-fold upper range K 46.8 pg/mL M 102.6 pg/mL		8/167 (4.79%)	75/407 (18.43%)	123/389 (31.62%)	92/211 (43.6%)	**<0.0001**	**0.0003 a** **<0.0001 b,c**	**0.0001 d** **<0.0001 e** **0.0276 f**
	**On discharge**	pg/mL	8.49 ± 28.59 0.8–337.0 (167)	100.38 ± 737.43 0.8–12,391.6 (407)	922.1 ± 9227.54 0.2–174,652.6 (389)	1770.07 ± 10,364.52 1.6–109,359.5 (211)	**0.0012**	0.06 a 0.208 **b** 0.068 c	0.299 d 0.093 **e** 0.753 f
**LDL-cholesterol** **(449)**	**On admission**	mg/dL	96.76 ± 37.36 35.0–209.0 (42)	104.01 ± 57.58 6.0–510.0 (136)	88.75 ± 41.81 23.0–230.0 (174)	76.22 ± 40.12 14.0–210.0 (97)	**0.0002**	0.775 a 0.617 **b** **0.024 c**	**0.048 d** **0.0001 e** 0.075 f
**HDL-cholesterol** **(451)**	**On admission**	mg/dL	43.28 ± 15.89 17.0–93.0 (43)	40.94 ± 17.34 2.0–120.0 (139)	38.91 ± 15.23 7.0–110.0 (172)	36.87 ± 13.41 8.0–79.0 (97)	0.071	N/A	N/A
**Triglycerides** **(639)**	**On admission**	mg/dL	212.22 ± 175.23 40.0–1100.0 (63)	178.25 ± 123.5 44.0–958.0 (213)	149.84 ± 84.96 50.0–586.0 (243)	126.78 ± 62.3 46.0–401.0 (120)	**<0.0001**	0.48 a **0.038 b** **0.002 c**	**0.026 d** **<0.0001 e** **0.019 f**
**Hormones**									
**25-hydroxy-vitamin D** (474)	**On admission**	ng/mL	26.95 ± 18.41 3.5–135.6 (62)	24.85 ± 17.66 3.5–146.1 (186)	23.25 ± 16.43 3.5–77.7 (173)	20.09 ± 14.66 3.5–65.5 (53)	0.1159	N/A	N/A
**TSH** **(820)**	**On admission**	mlU/L	1.33 ± 0.94 0.0–4.41 (106)	1.56 ± 2.47 0.01–28.81 (261)	1.48 ± 1.83 0.0–14.38 (299)	1.85 ± 3.71 0.0–38.24 (154)	0.2618	N/A	N/A

Continuous variables are presented as mean ± S, range (min–max), and number of non-missing values. Categorized variables are presented as percentages. Information about the numbers with valid values is provided in the left column. N/A: Not applicable.

### 3.3. Pharmacological Treatment during Hospitalization

#### 3.3.1. Medicines

Applied treatment during hospitalization between low-, moderate-, high-, and extreme-risk patients is presented in Table 5. Patients from the moderate risk cluster more often received systemic corticosteroids, convalescent plasma, Tocilizumab, and Remdesivir when compared to patients classified as low, high, and extreme risk. Often, antibiotic treatment has been observed in extreme-risk group patients.

#### 3.3.2. Treatment Procedures

Patients from the low-risk group rarely needed respiratory support during the hospitalization. On the contrary, patients from the high-risk stratum were expected for invasive ventilation more often than patients from other groups (Table 6). Moreover, patients in this group more often demanded therapy with catecholamines as well as hemodialysis.

### 3.4. Clinical Results

#### 3.4.1. VACO Risk Strata and Mortality Correlation

The correlation between VACO and mortality is collected in Table 7. Considerable differences regarding the in-hospital, three-month, and six-month mortality groups were observed. The mortality of patients in the VACO extreme-risk stratum increased over time and was 37.42%, 62.81%, and 78.44% for in-hospital, three-month, and six-month mortality groups, respectively. In the high-risk stratum, the mortality was 20.38%, 37.19%, and 58.77%. In the moderate-risk stratum, the mortality was 9.15%, 15.76%, and 35.49%. In the lower-risk stratum, the mortality was 2.12%, 2.69%, and 11.02%.

**Figure 1:**
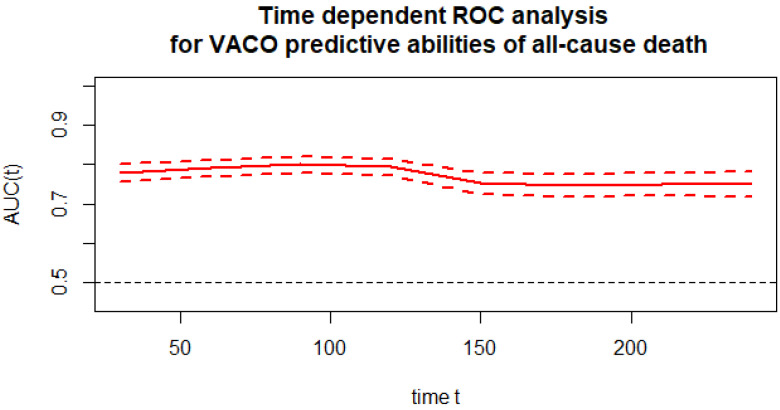
Time-dependent ROC analysis for the VACO predictive abilities of all-cause death.

The time-dependent ROC analysis (Figure 1) was used for the predictive power of the VACO risk strata for mortality at time t from hospital admission. All causes of death were considered in the analysis. The chart below shows the predictive ability expressed as the area under the ROC curve versus time along with the confidence intervals for that area. The time-dependent AUC for the VACO risk strata in predicting all-cause mortality was above 74.

**Figure 2:**
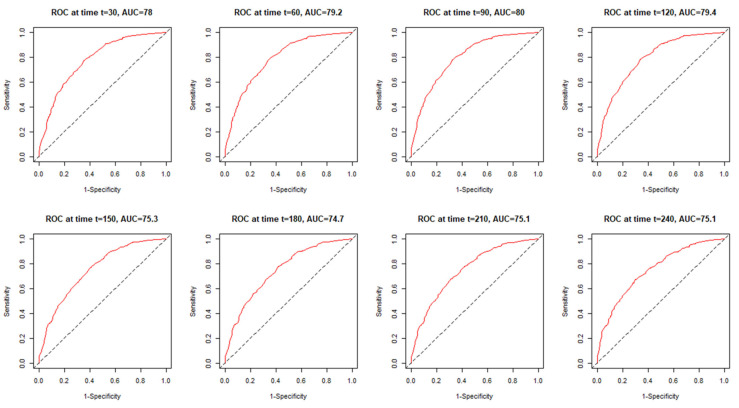
Time–ROC curves for the VACO risk strata in predicting total mortality.

Figure 2 presents the monthly time-dependent ROC (time–ROC) taking into account VACO. VACO maintained at a similar level with AUC ranging from 74.7 to 80.0.

**Figure 3:**
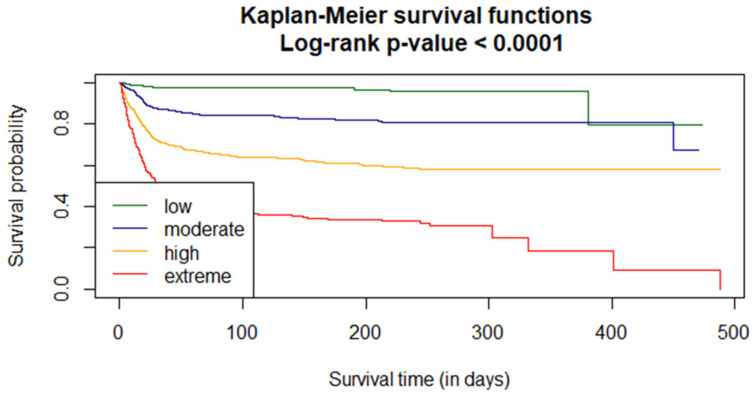
VACO risk strata analysis of the in-hospital probability for the COLOS population.

Survival curves were estimated using the Kaplan–Meier functions. The curves were compared using the Log-rank test. A *p*-value < 0.0001 indicates that the probability of survival in risk groups is significantly different (Figure 3).

The effect of the VACO risk strata stratification on COVID-19 mortality was analyzed using the Cox model. The proportionality coefficient in the Cox model is 1.0667 (with the standard deviation 0.00288). Change in the risk category increased death intensity by 1.0667. The 95% confidence interval for the ratio is 95% (CI 1.061–1.073).

#### 3.4.2. Secondary Outcomes and Its Correlation with the VACO Score

The clinical hospitalization events outcomes are pooled in Table 8. Patients from the extreme-risk stratum were more likely to develop cardiogenic shock, myocardial infarction, myocardial injury, stroke, pneumonia, acute kidney injury, and acute liver dysfunction. Patients from moderate-risk groups were more often admitted to ICU when compared to other patients. Patients from the high-risk group were more likely to develop septic shock. In the occurrence of aborted cardiac arrest, hypovolemic shock, venous thromboembolic disease, pulmonary embolism, complete respiratory failure, SIRS, multiple organ dysfunction syndrome, lactic acidosis, and non-significant differences were reported.

## 4. Discussion

The data used for this study clearly show that the VACO index demonstrated a great association between predictors and mortality. As was stated, age was the strongest predictor of mortality, reaching 44% among patients 90 or more years old [9]. Elderly patients were the most affected group by the COVID-19 pandemic. Studies conducted during and post-pandemic indicated that the severity of illness increases with age and the presence of certain comorbidities such as chronic kidney diseases, chronic obstructive pulmonary disease, coronary heart diseases, and respiratory diseases [15]. The mortality rate from the published papers increases with the advanced age (75–84 year old) when compared to the 5–17-year-old population. The likelihood of dying was 8700-fold higher for the elderly patients [16]. The mortality values predicted by the VACO scale presented in this scientific paper shows the dependence of risk stratification and mortality. Mortality increased with the risk stratum and advanced age. The in-hospital, three month, and six month mortality rates in the extreme risk patients group were 37.42%, 62.81%, and 78.44%, respectively; for comparison, the mortality rates for the low risk stratum were 2.12%, 2.69%, and 11%. The mortality values predicted by the C2HEST score in elderly subjects with COVID-19 published by Rola and Doroszko et al. [9] were 35.7%, 54.4%, and 65.9% for high-risk patients for in in-hospital, three-month, and six-month discharge groups, respectively. These predicted values by the C2HEST score were comparable to values predicted by the VACO index and presented in this article. Our previously published papers [17,18] state that the elderly population is more likely to be hospitalized or admitted to an intensive care unit and that advanced age has a positive correlation with increasing hospitalization time. In our study, patients from the extreme risk stratum had the longest hospitalization time when compared to patients from other risk groups. Moreover, patients from extreme risk groups were more often needed to be transferred to another hospital due to the worsening of their clinical condition. However, patients from the moderate-risk stratum were two times more often admitted to ICU than patients from the extreme-risk group. That is why it is critical to properly triage patients so that appropriate help is provided to patients at the right time. The VACO index seems to be a good example of a rating scale characterizing patients. The lack of knowledge of how SARS-CoV-2 affects people in different clinical conditions motived clinicians to adopt medical scales in order to triage COVID-19 patients. The reports presenting the usage of medical scales stated that the VACO (AUC of 0.740) scale has similar predictive performance compared with the mC2HEST (AUC of 0.809), C2HEST (AUC of 0.752), CHA2DS2-VASc (0.756), HATCH (0.722), and HAVOC (0.758) scores [19]. Due to the fact that SARS-CoV-2 is known to affect the respiratory system, parameters of ventilation are used worldwide in clinical practice to determine the severity of infection [20]. The application of the VACO index discussed in this study shows that respiratory rate values may not only influence the severity of disease progression. Respiratory rates compared between the groups were not found to be significant; nevertheless, patients from the extreme-risk group have the highest values of respiratory rate. The SpO_2_ in room air decreased with increasing group risk and was 89% for patients in the extreme-risk group. The elevated inflammatory biomarkers, namely CRP, interleukin-6, and interleukin-8, are related to the severity of the disease [21,22,23]. Moreover, patients suffering from long COVID syndrome are found to have elevated levels of IL-6, CRP, and TNF-α even up to months after the recovery [24]. The CRP levels measured on admission and on discharge for patients in the extreme-risk stratum were 90.02 mg/L and 85.25 mg/L, respectively, while CRP levels for patients in the low-risk stratum were 49.79 and 29.96 measured on admission and on discharge, respectively. It can be noted that the difference between CRP levels measured on admission and on discharge decreases as the VACO risk stratum increases. Our study indicates that there are more clinical factors in which VACO seems to be useful. The risk of incident atrial fibrillation increased significantly with a higher VACO score and was valued at 39.88% for patients in the extreme-risk stratum. The patients classified as in the extreme-risk stratum also had the highest values of cardiac biomarkers (BNP, NT-proBNP, and troponin) among the patients from the low, moderate, and high risk stratum. Patients from the extreme-risk stratum were also found to have more abnormalities detected during physical examination than the patients from the other VACO risk stratum groups. Patients with the more severe disease tended to have the lowest level of hemoglobin and blood platelet count; on the other hand, these patients had higher potassium ion concentrations with coexisting elevated INR levels as well as a higher serum level of urea and creatine coexisting with lower eGFR and albumin values. Henry et al. also concluded in a meta-analysis that “patients with severe and fatal disease had significantly increased WBC, and decreased lymphocyte and platelet counts compared to non-severe disease and COVID-19 survivors” [25]. The VACO index was also adopted to investigate the correlation with other comorbidities. The scientists validated the results on patients with hypertension and alcoholism. However, no statistical significance was recognized between hypertension and mortality factors [26]. The practical usage of the VACO may improve primary and booster vaccination prioritization. Moreover, the highest consciousness can be presented for individuals testing positive for SARS-CoV-2.

## 5. Conclusions

VACO is an efficient tool for clinical outcome and mortality prediction for patients admitted to hospital with proven SARS-CoV-2 infection. The usage of the VACO index, combined with an appropriate well defined medical interview and past medical history, allows the assumption that it can be a helpful instrument for the prediction of pre-hospital risk and for appropriate patient triaging, shortening the time from admission to diagnosis.

## 6. Limitations

The clinical outcome may be affected by a single-center registry result and retrospective analysis of the results. Moreover, some clinical data provided at the admission to the hospital and baseline laboratory assays conducted during the hospital stay may be incomplete, causing difficulty in the proper interpretation of the results.

## Figures and Tables

**Table 5:** COLOS population pharmacological treatment applied during hospitalization in the studied cohort.

Variables, Units (N)	Lower Risk (L) [0–0.292%]	Moderate Risk (M) [0.293–8.784%]	High Risk (H) [8.785–20.174%]	Extreme Risk (E) [>20.174%]	OMNIBUS *p-*Value	*p-*Value (for Post-Hoc Analysis)	
	n/N (% of Risk Category)	n/N (% of Risk Category)	n/N (% of Risk Category)	n/N (% of Risk Category)		a—L vs. M b—L vs. H c—L vs. E	d—M vs. H e—M vs. E f—H vs. E
**Applied Treatment and Procedures**							
**Systemic corticosteroid** (2183)	203/519 (39.11%)	402/710 (56.62%)	333/628 (53.03%)	158/326 (48.47%)	**<0.0001**	**<0.0001 a,b** 0.0553 c	1.0 d 0.1043 e 1.0 f
**Convalescent plasma** (2183)	45/519 (8.67%)	91/710 (12.82%)	76/628 (12.1%)	27/326 (8.28%)	**0.0361**	0.1623 a 0.4442 b 1.0 c	1.0 d 0.2552 e 0.5426 f
**Tocilizumab** (2183)	5/519 (0.96%)	15/710 (2.11%)	4/628 (0.64%)	1/326 (0.31%)	**0.0346**	1.0 a,b,c	0.2069 d 0.1733 e 1.0 f
**Remdesivir** (2183)	66/519 (12.72%)	141/710 (19.86%)	100/628 (15.92%)	36/326 (11.04%)	**0.0004**	**0.0075 a** 0.8787 b 1.0 c	0.4328 d **0.0039 e** 0.3089 f
**Antibiotic** (2183)	203/519 (39.11%)	413/710 (58.17%)	396/628 (63.06%)	228/326 (69.94%)	**<0.0001**	**<0.0001 a,b,c**	0.4613 d **0.0023 e** 0.2436 f

Categorized variables are presented as percentages. Information about the numbers with valid values is provided in the left column.

**Table 6:** Applied treatment and procedures.

Variables, Units (N)	Lower Risk (L) [0–0.292%]	Moderate Risk (M) [0.293–8.784%]	High Risk (H) [8.785–20.174%]	Extreme Risk (E) [>20.174%]	OMNIBUS *p-*Value	*p-*Value (for Post-Hoc Analysis)	
	Mean ± SD Min–Max (N) or n/N (% of Risk Category)	Mean ± SD Min–Max (N) or n/N (% of Risk Category)	Mean ± SD Min–Max (N) or n/N (% of Risk Category)	Mean ± SD Min–Max (N) or n/N (% of Risk Category)		a—L vs. M b—L vs. H c—L vs. E	d—M vs. H e—M vs. E f—H vs. E
**Applied Treatment and Procedures**							
**Respiratory support applied during the hospitalization** **no oxygen** (2183)	344/519 (66.28%)	319/710 (45.06%)	259/628 (41.31%)	110/326 (33.74%)	**<0.0001**	**<0.0001 a,b,c**	0.4313 d **<0.0001 e** **0.0071 f**
**low flow oxygen support**	131/519 (25.24%)	256/710 (36.16%)	229/628 (36.52%)	147/326 (45.09%)			
**high flow nasal cannula** **non-invasive ventilation**	18/519 (3.47%)	39/710 (5.51%)	39/628 (6.22%)	35/326 (10.74%)			
**invasive ventilation**	25/519 (4.82%)	85/710 (12.01%)	78/628 (12.44%)	24/326 (7.36%)			
**Oxygenation parameters from the period of qualification for advanced respiratory support**: SpO_2_, % (631)	93.68 ± 4.68 70–100 (189)	88.77 ± 8.61 50–100 (180)	86.01 ± 9.85 55–99 (160)	85.77 ± 9.92 59–99 (102)	**<0.0001**	**<0.0001 a,b,c**	**0.034 d** 0.056 e 0.998 f
**Therapy with catecholamines** (2183)	25/519 (4.82%)	75/710 (10.56%)	80/628 (12.74%)	38/326 (11.66%)	**<0.0001**	**0.0025 a** **<0.0001 b** **0.0023 c**	1.0 d,e,f
**Coronary revascularization** **or/and an indication for coronary revascularization** (2183)	1/519 (0.19%)	5/710 (0.7%)	12/628 (1.91%)	12/326 (3.68%)	**<0.0001**	1.0 a 0.052 b **0.0005 c**	0.3237 d **0.0058 e** 0.7537 f
**Hemodialysis** (2183)	4/519 (0.44%)	25/710 (3.52%)	32/628 (5.1%)	10/326 (3.07%)	**0.0007**	**0.0192 a** **0.0004 b** 0.1395 c	1.0 d,e,f

Continuous variables are presented as mean ± SD, range (min–max), and number of non-missing values. Categorized variables are presented as percentages. Information about the numbers with valid values is provided in the left column.

**Table 7:** Total vs. in-hospital mortality in the VACO risk strata of the COLOS study cohort.

Variables, Units (N)	Lower Risk (L) [0–0.292%]	Moderate Risk (M) [0.293–8.784%]	High Risk (H) [8.785–20.174%]	Extreme Risk (E) [>20.174%]	OMNIBUS *p-*Value	*p-*Value (for Post-Hoc Analysis)	
	n/N (% of Risk Category)	n/N (% of Risk Category)	n/N (% of Risk Category)	n/N (% of Risk Category)		a—L vs. M b—L vs. H c—L vs. E	d—M vs. H e—M vs. E f—H vs. E
**All-cause mortality rate**							
**In-hospital mortality** (2183)	11/519 (2.12%)	65/710 (9.15%)	120/628 (20.38%)	122/326 (37.42%)	**<0.0001**	**<0.0001 a,b,c**	**<0.0001 d,e,f**
**3-month mortality** (2087)	13/483 (2.69%)	107/679 (15.76%)	225/605 (37.19%)	201/320 (62.81%)	**<0.0001**	**<0.0001 a,b,c**	**<0.0001 d,e,f**
**6-month mortality** (1174)	13/118 (11.02%)	115/324 (35.49%)	238/405 (58.77%)	211/269 (78.44%)	**<0.0001**	**<0.0001 a,b,c**	**<0.0001 d,e,f**

Continuous variables are presented as mean ± SD, range (min–max), and number of non-missing values. Categorized variables are presented as a number with a percentage. Information about the numbers with valid values is provided in the left column.

**Table 8:** Clinical non-fatal events and hospitalization results of the VACO risk of the COLOS cohort study.

Variables, Units (N)	Lower Risk (L) [0–0.292%]	Moderate Risk (M) [0.293–8.784%]	High Risk (H) [8.785–20.174%]	Extreme Risk (E) [>20.174%]	OMNIBUS *p-*Value	*p-*Value (for Post-Hoc Analysis)	
	Mean ± SD Min–Max (N) or n/N (% of Risk Category)	Mean ± SD Min–Max (N) or n/N (% of Risk Category)	Mean ± SD Min–Max (N) or n/N (% of Risk Category)	Mean ± SD Min–Max (N) or n/N (% of Risk Category)		a—L vs. M b—L vs. H c—L vs. E	d—M vs. H e—M vs. E f—H vs. E
**Hospitalization**							
**Duration of hospitalization, days** (2183)	7.74 ± 8.7 1–59 (519)	13.05 ± 14.84 1–131 (710)	14.55 ± 15.48 1–124 (628)	14.55 ± 14.41 1–121 (326)	**<0.0001**	**<0.0001 a,b,c**	0.274 d 0.415 e 1.0 f
**Admission at intensive care unit** (2183)	30/519 (5.78%)	88/710 (12.39%)	75/628 (11.94%)	22/326 (6.75%)	**<0.0001**	**0.0009 a** **0.0028 b** 1.0 c	1.0 d 0.0511 e 0.0971 f
**End of hospitalization** **death** (2183)	11/519 (2.12%)	65/710 (9.15%)	128/628 (20.38%)	122/326 (37.42%)	**<0.0001**	**<0.0001 a,b,c**	**<0.0001 d,e,f**
**discharge to home** **full recovery**	447/519 (86.13%)	459/710 (64.65%)	297/628 (47.29%)	113/326 (34.66%)			
**transfer to another hospital—worsening**	26/519 (5.01%)	84/710 (11.83%)	104/628 (16.56)	66/326 (20.25)			
**transfer to another hospital—in recovery**	35/519 (6.74%)	102/710 (14.37%)	99/628 (15.76%)	25/326 (7.67%)			
**Aborted cardiac arrest** (2183)	3/519 (0.58%)	8/710 (1.13%)	8/628 (1.27%)	5/326 (1.53%)	0.5334	N/A	N/A
**Shock** (2183)	17/519 (3.28%)	65/710 (9.15%)	70/628 (11.15%)	35/326 (10.74%)	**<0.0001**	**0.0004 a** **<0.0001 b** **0.0001 c**	1.0 d,e,f
**Hypovolemic shock** (2183)	5/519 (0.96%)	12/710 (1.69%)	11/628 (1.75%)	7/326 (2.15%)	0.556	N/A	N/A
**Cardiogenic shock** (2183)	2/519 (0.39%)	5/710 (0.7%)	9/628 (1.43%)	16/326 (4.91%)	**<0.0001**	1.0 a 0.7463 b **<0.0001 c**	1.0 d **0.0002 e** **0.0138 f**
**Septic shock** (2183)	13/519 (2.5%)	52/710 (7.32%)	56/628 (8.92%)	19/326 (5.83%)	**0.0001**	0.0019 a **<0.0001 b** 0.1362 c	1.0 d,e 0.7294 f
**Venous thromboembolic disease** (2183)	12/519 (2.31%)	28/710 (3.94%)	18/628 (2.87%)	11/326 (3.37%)	0.4122	N/A	N/A
**Pulmonary embolism** (2183)	10/519 (1.93%)	22/710 (3.1%)	16/628 (2.55%)	11/326 (3.37%)	0.7591	N/A	N/A
**Myocardial infarction** (2183)	0/519 (0.0%)	3/710 (0.42%)	11/628 (1.75%)	12/326 (3.68%)	**<0.0001**	1.0 a **0.0086 b** **<0.0001** c	0.166 d **0.0008 e** 0.4564 f
**Myocardial injury** (1174)	8/167 (4.79%)	75/407 (18.43%)	123/389 (31.62%)	92/211 (43.6%)	**<0.0001**	**0.0003 a** **<0.0001 b,c**	**0.0001 d** **<0.0001 e** **0.0276 f**
**Acute heart failure** (2183)	0/519 (0.0%)	11/710 (1.55%)	20/628 (3.18%)	45/326 (13.8%)	**<0.0001**	0.0662 **a** **0.0006 b** **<0.0001 c**	0.4289 d <**0.0001 e,f**
**Stroke/TIA transient ischemic attack** (2183)	0/519 (0.0%)	9/710 (1.27%)	18/628 (2.87%)	17/326 (5.21%)	**<0.0001**	0.1522 a **0.0016 b** **<0.0001** c	0.3601 d **0.0022 e** 0.5955 f
**New cognitive signs and symptoms** (2183)	0/519 (0.0%)	16/710 (2.25%)	55/628 (8.76%)	50/326 (15.34%)	**<0.0001**	**0.0086 a** **<0.0001 b,**c	**<0.0001** d,e **0.0178 f**
**Pneumonia** (2183)	131/519 (25.24%)	385/710 (54.23%)	348/628 (55.41%)	197/326 (60.43%)	**<0.0001**	**<0.0001 a,b,c**	1.0 d 0.4297 e 0.9413 f
**Complete respiratory failure** (276)	5/14 (37.71%)	34/72 (47.22%)	55/104 (52.88%)	52/86 (60.47%)	0.2064	N/A	N/A
**SIRS systemic inflammatory response syndrome** (2114)	50/474 (10.55%)	74/692 (10.69%)	56/625 (8.96%)	40/323 (12.38%)	0.4188	N/A	N/A
**Sepsis** (884)	1/240 (0.42%)	6/275 (2.18%)	7/227 (3.08%)	9/142 (6.34%)	0.0047	0.7711 a 0.1981 b **0.0047** c	1.0 d 0.2901 e 1.0 f
**Acute kidney injury** (2183)	12/519 (2.31%)	71/710 (10.0%)	94/628 (14.97%)	59/326 (18.1%)	**<0.0001**	**<0.0001 a,b,c**	**0.0448 a** **0.0023 b** 1.0 c
**Acute liver dysfunction** (1974)	3/423 (0.71%)	25/651 (3.84%)	16/598 (2.68%)	22/302 (7.28%)	**<0.0001**	**0.019 a** 0.2392 b **<0.0001** c	1.0 d **0.002 e** **0.0128 f**
**Multiple organ dysfunction syndrome** (2183)	4/519 (0.77%)	15/710 (2.11%)	11/628 (1.75%)	7/326 (2.15%)	0.282	N/A	N/A
**Lactic acidosis** (245)	1/13 (7.69%)	3/60 (5.0%)	7/95 (7.37%)	11/77 (14.29%)	0.2598	N/A	N/A
**Bleeding** (2183)	18/519 (3.47%)	32/710 (4.51%)	38/628 (6.05%)	26/326 (7.98%)	**0.0201**	1.0 a 0.3585 b **0.0402** c	1.0 d 0.2094 e 1.0 f

Continuous variables are presented as mean ± SD, range (minimum–maximum), and number of non-missing values. Categorized variables are presented as percentages. Information about the numbers with valid values is provided in the left column. Abbreviations: OMNIBUS—analysis of variance, N—valid measurements, n—number of patients with a parameter above cut-off point, SD—standard deviation.

## Data Availability

The datasets used and/or analyzed during the current study are available from the corresponding author upon reasonable request.
